# The Quality of Life, Psychological Health, and Occupational Calling of Korean Workers: Differences by the New Classes of Occupation Emerging Amid the COVID-19 Pandemic

**DOI:** 10.3390/ijerph17165689

**Published:** 2020-08-06

**Authors:** Young-Jae Kim, Seung-Woo Kang

**Affiliations:** Department of Physical Education, Chung-Ang University, Seoul 06974, Korea; yjkim@cau.ac.kr

**Keywords:** COVID-19, quality of life, psychological health, occupational calling, CASP-19, COVID-19 new classes of occupation, work environment

## Abstract

This study aimed to create new classifications for occupations that have emerged from the COVID-19 pandemic in Korea, based on Reich’s classifications for the United States. We examined Korean workers’ occupational calling, psychological health, and quality of life. An online questionnaire was administered and data from 1029 Korean workers were analyzed. The questionnaire comprised the Korean version of the Multidimensional Calling Measure to assess occupational calling, the Psychosocial Well-being Index-short form for psychological health, and the Control, Autonomy, Self-realization, and Pleasure (CASP-19) scale for quality of life. We created a Korean-adapted version of the classes of occupation based on those created by the COVID-19 situation in the USA. Our results showed that Korean workers had a high perceived calling to work, and different classes showed different levels of quality of life and psychological health. We need a health concentration management system for essential groups or personal safety protection equipment should be provided. Education on infection control should be offered and effective medical system processes should be in place. We need to develop technology to respond to medical needs online, remotely, or telephonically. The government should implement policies to ensure job security and to improve wages and welfare.

## 1. Introduction

COVID-19 has had, and continues to have, tremendous negative economic and social impacts in multiple countries worldwide [1,2]. In particular, in an attempt to cut operation costs, firms around the world have forced workers into furlough and layoffs; these actions have increased job insecurity, which has turned into growing anxiety and psychological stress [3].

Owing to the pandemic, most people are experiencing different work schedules and routines compared to their pre-COVID-19 work chores, and this adaptation has brought about changes in various occupations, as well as new ones [4]; thus, an American professor, Robert Reich, has presented new classes of occupation that have emerged from the COVID-19 pandemic [4], which are classified by work routines and payment changes. These are the Remotes, the Essentials, the Unpaid, and the Forgotten. After this general introduction to the current Korean and American situation, let us conceptualize Reich’s [4] classifications and provide more information on how we have applied these classifications to the Korean setting.

The Remotes are those with stable occupations that enable them to continue working remotely despite the pandemic; these also exist in Korea, and their work is characterized by work environments that can be altered anytime and anywhere, thus enabling them to work remotely with effectiveness through the use of advanced smart equipment [5].

The Essentials are those who provide work that is essential even during a pandemic situation, thus their work lives have not changed substantially compared to their pre-pandemic work lives (e.g., field and social services workers). The existing classification of occupations does not classify this class according to altered work hours [4,5,6]. In Korea, the entire work market was able to quickly adapt to this crisis environment by implementing remote work systems, which thereby allowed for increased work flexibility across the labor market [7]. The Essentials are classified as Essentials-1, who work just as they did before the pandemic, without wage changes, but with shortened work hours or working every other day; and Essentials-2, who work shorter hours or every other day, without wage changes, with work routines similar to their pre-pandemic routines.

The Unpaid are those who continue to go to work in a manner similar to their pre-pandemic states, but at a reduced pay (i.e., owing to diminished workdays or hours), and those who are not currently working. In the USA, this class includes furloughed workers and laid-off workers [4]. Regarding conceptualization, the Unpaid differ from the Essentials in that they have different wages compared to their pre-pandemic wages (e.g., only a base salary or no salary is paid). Similar to the USA, Korea has been economically impacted by the health crisis related to the COVID-19 pandemic; thus, companies nationwide have stopped operating either partially or in full, leading contractors, subcontractors, and independent services [8] to have had reduced monthly incomes owing to reduced work hours. However, in Korea, this classification needs to be adapted as there are two clear situations that differ; thus, we have included the Unpaid-1, who are those that continue to go to work as before, but with reduced wages; and the Unpaid-2, who work shorter hours or go to work every other day and also experience reduced wages.

Finally, the Forgotten are all others for whom social distancing is practically impossible, including prison inmates, those in immigration detention centers, those in camps for migrant farmworkers, people in reservations (in the original classification, these were referred to as Native American in reservations [4]), and homeless people in shelters. However, these structures are based on American society, and compared to the Korean population structure, there are clear differences [9]. Korea has no indigenous peoples to shelter and few centers or camps related to immigration and migration. Moreover, there are prisoners and homeless facilities, but not as many as in the United States. Therefore, this part was left out, and we classified it as a general occupational class. Thus, this class of occupation also needs to be adapted to the Korean setting. In our revised version, the Forgotten in Korea differs from the United States, which helps to solve unpaid leave, absences, situational conditions, and situations that had not existed before and thus could not be foreseen. The six groups that were forgotten include those on unpaid leave, leave of absence, work standby status, and those in situations in which they are not currently working.

Previous studies have shown that a stable job increases workers’ quality of life (QoL) and also helps individuals protect themselves from misfortunes in society [10]. However, in the Korean setting, people accept working for the greater good of their country even amid a national disaster that threatens their QoL, principally because they deem this situation as their “fate” [11]; that is, Korean people show a high occupational calling, and it is so high that, even during a crisis, they prioritize work over their QoL [12,13]. However, as aforementioned, many Korean workers have been facing unstable employment situations owing to the recent COVID-19 pandemic [14,15]. Notwithstanding this fact, Korean companies and local governments have been able to implement flexible work schedules, video conferences, and work-from-home conditions for most occupations, and these measures have, consequently, diminished the risk for the more severe impacts of the pandemic on the country [16]. Nevertheless, worldwide, COVID-19 has had an adverse impact not only on individuals’ mental health, but also on their overall QoL [17,18,19]. Thus, there seems to be a need for an examination of the impact of the COVID-19 pandemic on Korean workers’ work routines, wages, sense of calling, psychological health, and QoL (Table 1).

In Korea, workers are striving and trying their best to overcome this health crisis that has brought different consequences to various sectors of society. Therefore, it can be said that Korean workers have a high occupational calling amid this national crisis. However, there was no classification or reference to occupational calling offered by Reich [4]. Occupational calling means a sense of consciousness toward their work and an attitude that gives life meaning and purpose to life and activities as a worker [20]. Workers higher in occupational calling are typically dedicated to the achievement of their work goals [20,21], and further, they showed higher levels of engagement when faced with stressors that can be addressed through increased effort. Therefore, this dedication to their work leads to the positive professional ability between challenge stressors and engagement in workers higher in calling. However, a study in the United States showed that individuals with a high calling were more sensitively responsive to barriers that interfere with their purposes [22]. Through these things, Korean workers seem to have a relatively high calling in that they continue to work throughout social difficulty, that is, during a national disaster. In the United States, the response to the pandemic has forced the country into a rapid economic depression, mainly because many of the services in the country require people to physically go into the services in order to access them; thus, they cannot be converted to remote operations (e.g., retail stores, restaurants, and hospitality industries).

Furthermore, mass layoffs have ensued as a consequence of efforts to reduce expenditure, and these are spreading to the news industry, to tech companies, and to manufacturers of consumer goods [2,23]. As shown in this study, while the situation in the USA seems similar to that in Korea, the broad spread of COVID-19 among the workforce has prolonged the hindrances to a resumption of normal work routines [24]. In Korea, survey results have shown that the proportion of non-regular workers who experienced unemployment after COVID-19 was more than six times that of regular workers [25,26]. Therefore, it seems inappropriate to compare the classes of occupation that are emerging in the USA due to COVID-19 with those emerging in Korea. Thus, this study aimed to implement the Korean version of the COVID-19 classes of occupation by referencing the existing classifications whilst examining the psychological states of Korean workers. Consequently, this study is designed to reflect the current Korean situation in order to examine the sense of occupational calling, psychological health, and quality of life of Korean workers during COVID-19. We intend to present our basic data in the hope that it will be of national assistance in the preparation of potential future disasters.

## 2. Materials and Methods

### 2.1. Sample and Participants

The study participants were Korean office workers. A research company (Panel Now) administered a survey online from May 25 to 30, 2020. We collected each participant’s current occupational calling, quality of life, and psychological health since January 2020 when COVID-19 occurred. A total of 1426 subjects were obtained through multi-tiered cluster sampling, and 1167 questionnaires were collected, a recovery rate of 77.8%. Part 1029 was used for final analysis. This study was approved by the institutional review board (IRB) at Chung-Ang University (1041078-202005-HRSB-117-01), and we declare that the investigations were carried out following the rules of the Declaration of Helsinki of 1975, revised in 2013. Additionally, all subjects gave their informed consent for inclusion before they participated in the study.

### 2.2. Measurements

A cross-sectional survey was chosen to assess the occupational calling, psychological health, and QoL of workers in Korea amid the COVID-19 pandemic. For such purposes, the questionnaire scale enquired into their social background and their leisure activities (gender, age, marital status, salary). The Korean version of the Multidimensional Calling Measure (MCM-K), the Psychosocial Well-being Index short form (PWI-SF), and the Control, Autonomy, Self-realization, and Pleasure (CASP-19) scale were used. Furthermore, the questionnaires were administered based on our version—adapted to the Korean setting—of Reich’s classes of occupation that arose due to the COVID-19 pandemic [4].

#### 2.2.1. MCM-K

In order to assess the concept of occupational “calling”, we utilized the MCM-K, which is the Korean-adapted version [27] of the MCM, which was developed from the English versions of Hagmaire and Able [28]. It contains nine items, and each is rated on a 5-point Likert scale. The MCM-K was shown to have adequate levels of reliability and validity among Korean workers [27]; in our study, we summed the scores for all items to compute the total score. The Cronbach’s α of the original tool was 0.90; in this study, α = 0.818.

#### 2.2.2. PWI-SF

In order to assess psychological health, we utilized the PWI-SF, which is the Korean-adapted version of the GHQ-60, developed by Goldberg [29,30]. It contains 18 items and each is rated on a 4-point Likert scale (0 = never true; 1 = sometimes true; 2 = mostly true; 3 = always true); in our study, we summed the scores for all items to compute the total score. That is, the higher the score, the lower the psychological health. The Cronbach’s α of the original tool was 0.867; in this study, α = 0.830.

#### 2.2.3. CASP-19

In order to assess QoL, we utilized the CASP-19 developed by Higgs, Paul et. al. [31] It contains 19 items and four factors: Control (4 items), Autonomy (5 items), Pleasure (5 items), and Self-realization (5 items); each item is rated on a 4-point Likert scale (0 = never true; 1 = sometimes true; 2 = mostly true; 3 = always true). In our study, we summed the scores for all items to compute the total score. The Cronbach’s α of the original tool was 0.90; in this study, α = 0.886.

### 2.3. Data Analysis

SPSS for Windows (Version 25.0, IBM, Armonk, NY, USA) was used for data analysis. We used frequency analysis for sociodemographic characteristics, and reliability analysis to check the reliability of the scales. Additionally, we used one-way ANOVA (Analysis of variance) with Scheffé post-hoc tests and Correlation Analysis to assess QoL, psychosocial health, and the calling of Korean workers, using our Korean-adapted version of the new classes of occupation.

## 3. Results

### 3.1. Participants’ Calling, Psychological Health, and QoL Levels by Sociodemographic Characteristics

Table 2 shows participants’ occupational calling, psychological health, and QoL by their sociodemographic characteristics. First, the mean occupational calling score was 31.20 in males (*n* = 546, 53.1%, F = 16.44, *P* = 0.001) and 29.92 in female workers (*n* = 483, 46.9%, F = 16.44, *P* = 0.001); thus, male workers had a higher perceived calling. Additionally, the mean psychological health and QoL scores of male workers were 22.13 (F = 8.73, *P* = 0.01) and 31.50 (F = 21.50, *P* = 0.001), respectively; for female workers, they were 23.42 (F = 8.73, *P* = 0.01) and 29.02 (F = 21.50, *P* = 0.001), respectively; thus, male workers had better psychological health and a higher QoL.

Moreover, workers in their 50s (*n* = 108, 10.5%) had the highest occupational calling score (M = 32.02, F = 6.06, *P* = 0.001), while workers in their 30s (*n* = 559, 54.3%) had the lowest calling score (M= 29.96). In terms of marital status, the married workers (*n* = 543, 52.8%, F = 7.24, *P* = 0.01) showed a mean calling score of 31.56, a mean psychological score of 21.96, and a mean QoL score of 31.40 (F = 9.01, *P* = 0.001). In terms of monthly income, the 10 million KRW (Korean Won) or higher group (*n* = 18, 1.7%) showed a mean calling score of 34.33 (F = 13.35, *P* = 0.001), a mean psychological score of 19.39 (F = 5.55, *P* = 0.001), and a mean QoL score of 35.39 (F = 9.66, *P* = 0.001).

Additionally, the most common leisure activities among participants were resting (*n* = 891, 86.6%) and enjoying hobbies and entertainment (*n* = 601, 58.4%); however, we found no significant effects of these two on calling, psychological health, or QoL. The mean calling score among participants who chose watching cultural and art activities (*n* = 562, 54.6%) was 31.10 (F = 11.92, *P* = 0.001); additionally, the mean occupational calling and QoL scores of those who chose participating in cultural and art activities (*n* = 142, 13.8%) were 32.01 (F = 12.92, *P* = 0.001) and 32.46 (F = 10.02, *P* = 0.001), respectively. The mean occupational calling scores were similar for those who chose watching sports (*n* = 421, 40.9%, *M* = 31.00, F = 4.53, *P* = 0.05), going on tourism activities (*n* = 514, 50.0%, *M* = 31.19, F = 14.24, *P* = 0.001), and participating in social and other activities (*n* = 324, 31.5%, *M* = 31.10, F = 4.68, *P* = 0.01).

### 3.2. New Occupational Class, Calling, Psychological Health, and QoL due to COVID-19 Epidemic, and Correlation Analysis

First, there was a significant negative correlation between new occupational class and QoL, and a positive correlation between new occupational class and psychological health. However, there was no correlation with occupational calling and new occupational class. The correlation between QoL and occupational calling was negative. Finally, in the case of QoL, there was a significant negative correlation with occupational calling (Table 3).

### 3.3. Korean-Adapted New Classes of Occupation, Differences in Calling, Psychological Health, and QoL

**Figure 1:**
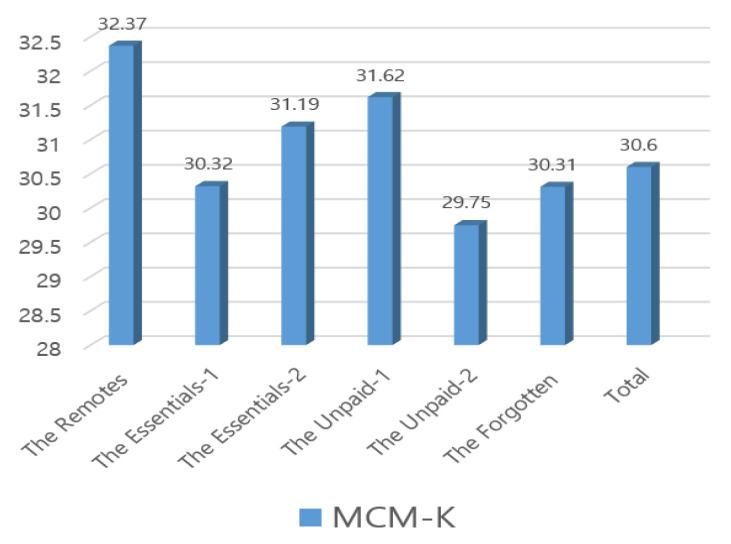
MCM-K(Multidimensional Calling Measure), Participants’ occupational calling by our Korean-adapted version of the new classes of occupation owing the COVID-19 pandemic.

**Figure 2:**
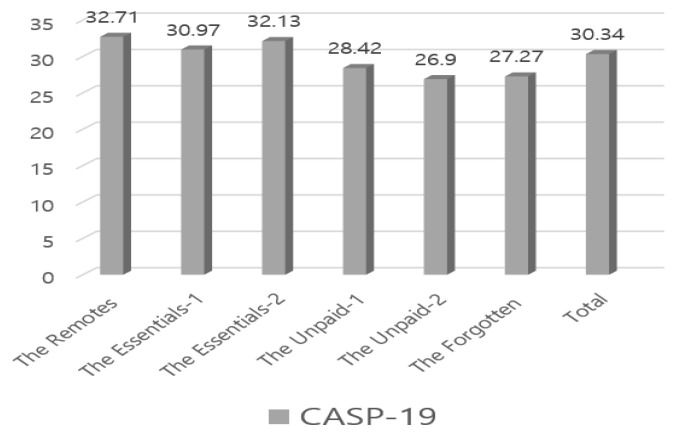
CASP-19 (Control, Autonomy, Self-realization, and Pleasure), Participants’ quality of life by our Korean-adapted version of the new classes of occupation owing the COVID-19 pandemic.

**Figure 3:**
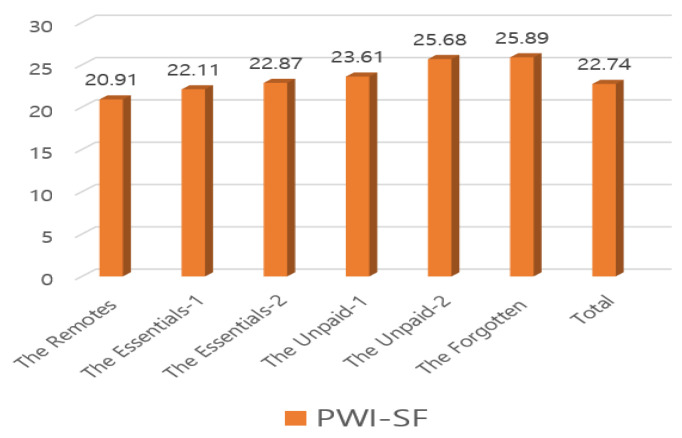
(PWI-SF, Psychosocial Well-being Index short form) Participants’ psychological health by our Korean-adapted version of the new classes of occupation owing the COVID-19 pandemic.

Both Table 4 and Figure 1, Figure 2 and Figure 3 show participants’ occupational calling, psychological health, and QoL levels from our Korean-adapted version of the new classes of occupation. The Remotes (*n* = 68, 6.6%) had the highest scores for all variables: occupational calling (*M* = 32.37, F = 3.53, *P* = 0.01), psychological health (*M* = 20.91, F = 7.98, *P* = 0.001), and QoL (*M* = 32.71, F = 7.54, *P* = 0.001). Regarding occupational calling scores, the Remotes were followed by the Unpaid-1 (*M* = 31.62, *n* =108, 10.5%) and the Essentials-2 (*M* = 31.19, *n* = 53, 5.2%); regarding the psychological health score, the Remotes were followed by the Essentials-1 (*M* = 22.11, *n* = 657, 63.8%) and the Essentials-2 (*M* = 22.87, *n* = 53, 5.2%). Regarding QoL, the Remotes were followed by the Essentials-2 (*M* = 32.13, *n* = 53, 5.2%) and the Essentials-1 (*M* = 30.97, *n* = 657, 63.8%). Conversely, the Unpaid-2 (*n* = 68, 6.6%) had low occupational calling (*M* = 29.75) and QoL (*M* = 26.90) scores, and the Forgotten (*n* = 75, 7.3%) had the lowest psychological health scores (*M* = 28.11).

## 4. Discussion

First, Korean workers’ occupational calling, psychological health, and QoL levels differed by sex: male workers had higher levels overall when compared to female workers, who had lower levels overall. We speculate that these results appeared owing to the Korean culture; Korea has long been influenced by the patriarchal Confucian culture [32], which follows the traditional ideological custom of presumed male superiority. Confucian patriarchal structure continues to have a great influence on the Korean worker of today, thus the social behavior pattern of Koreans refers to the Confucian social structure in which women are dominated by men [33]. This has had an impact on the percentage of women employed, mainly owing to the economic depression brought upon by the COVID-19 pandemic, thereby leading to further social disparity and reduced employment opportunities [23]. Therefore, stakeholders related to work equity should endeavor to develop precautionary interventions aimed at balancing employment opportunities for Korean men and women during national disasters, such as the current pandemic.

Second, Korean workers’ occupational calling, psychological health, and QoL differed by marital status: those who were married showed higher levels overall to those who were single. Namely, marriage seems to greatly impact Korean workers’ QoL and psychological health financially and psychologically. These results corroborate a previous study analyzing similar variables [34,35].

Third, the Remotes had the highest occupational calling levels, whereas the Unpaid-2 had the lowest. The Remotes and Unpaid-1 (who showed high calling scores) encompass people that principally work from home and who work to stabilize society (e.g., physicians, nurses, civil servants) [36,37]. Previous studies have shown that workers in these types of occupations often make sacrifices for the benefit of public motivation, and that workers in the public and health sectors (which relate to the occupations included in the Remotes and Unpaid-1 classifications) exhibit higher levels of occupational calling [38,39], thereby corroborating our findings. For essential workers, and especially healthcare workers, quality of life and psychological wellbeing could be affected by the stressful and traumatic events experienced in the work context [40,41]. Thus, our results, and the current literature, suggest that Korean workers indeed have a strong sense of occupational calling, in that they sacrifice themselves to help others with the intention of overcoming the national crisis through their high civic awareness. Furthermore, Korea’s professional background is highly influenced by personal values, perceptions, and pride, which is supported by existing literature [42,43]. In other words, it is judged that vocational education to increase job consciousness, such as conversation and mental recovery, patience, courage, and self-direction, is always necessary.

Fourth, the results for participants’ differences in psychological health were very similar to those for occupational calling. Namely, the Remotes, Essentials-1, and Essentials 2-, whose employment status or economic situation were not altered by COVID-19, exhibited greater psychological health than others and the differences between these classes were not significant; these findings are consistent with previous studies [44,45]. In other words, the more stable that workers feel their jobs are, the higher their occupational calling, which in turn showed that they are in good psychological health. Conversely, the Forgotten and the Unpaid-2 showed poorer psychological health levels, which may relate to their more unstable employment status owing to the pandemic (compared to the three aforementioned classes), and the toll that such instability takes on their personal financial situation; correlatively, several studies have provided similar results in this regard [46,47,48,49]. Therefore, Korean managers across all industries should endeavor to create disaster management schemes that ensure that working environments and employment opportunities remain stable even under such dire circumstances. Moreover, we suggest the development of intensive management systems aimed at the occupations that are directly threatened by such disasters. In other words, it is important to prepare for the occurrence of a national disaster with regard to Korean workers, particularly in crises that may cause unstable working environments and employment issues, such as a pandemic like COVID-19, in the future. Furthermore, a well-paid and quality work system is needed to work from home work remotely with effectiveness through the use of advanced smart equipment [6]. We need to ensure job security for non-regular, temporary, and day-to-day employees. In addition, groups who are unable to take a vacation due during a disaster should have a holiday, vacation, wage, or welfare system.

Finally, the Remotes showed the highest QoL. Conversely, although the Unpaid-2 were required to continue working during the COVID-19 pandemic, they had their wages reduced; such modifications may be related to their reduced QoL. This finding is corroborated by a previous study [50]. Correlatively, another study has reported that people’s QoL may decrease when they experience physical and mental pain, activity restrictions, and environmental exposure [51]. Moreover, a bulk of the literature [52,53,54,55,56,57] has shown the necessity of implementing measures to address the changes in Korean workers’ psychological health and QoL levels owing to the national crisis caused by the COVID-19 pandemic. Therefore, governmental support and various leisure activity programs should be devised to promote the QoL of the Unpaid-2 and the Forgotten, as even during the crisis these people will eventually return to their workplaces owing to financial reasons.

## 5. Conclusions

In summary, our results showed that: first, there were significant variations in our sample of Korean workers regarding their occupational calling, psychological health, and QoL levels by their sociodemographic characteristics. Second, there were significant variations in the occupational calling, psychological health, and QoL of our sample in the six new classes of occupation that emerged from the COVID-19 pandemic in Korea, as adapted from Reich’s [4] classifications.

Thus, we concluded that: first, being able to work from home may have helped Korean workers maintain their appropriate levels of QoL and psychological health during the COVID-19 pandemic. Second, and contrariwise, the Unpaid and the Forgotten showed lower levels of QoL and psychological health, thereby demonstrating an urgent need for resolutions, through governmental policies and various applicable programs aimed at helping these classes to cope with the current disastrous situation. Third, among the new COVID-19 classes of occupation, the Remotes showed the highest occupational calling levels; namely, the Remotes in Korea seem to have a high perceived occupational calling and aim to provide their best in their work. Therefore, to the extent to which it is possible, other classes of occupation should try to have a high occupational calling with pride in their careers, which may help them endure future crises.

In conclusion, this study confirmed the new occupational groups in Korean society that emerged during the COVID-19 pandemic, and further, found that these groups can reveal the self-perceived quality of life and psychological health of Korean workers. In the future, it is important to prepare for a national disaster with regard to workers who may experience unstable working conditions and employment issues caused by such a crisis. Therefore, we propose a contingency plan to overcome these issues. First, we need to provide the Essentials with the personal safety protection equipment that is directly needed due to the threat of COVID-19 through health-focused management systems, health care visits, health risk identification, and health counseling. Second, education on infection control should be offered and effective medical system processes should be in place, as high quality education practices are known to improve worker confidence or readiness [6]. Third, it is necessary to develop the technology to respond to medical needs online, remotely, or telephonically. The government should put policies in place to ensure job security and to improve wages and welfare. Lastly, for the essential class who cannot take vacations due to COVID-19, workers should at least be provided with living wages and welfare physiological expenses, including paid sick leave. Indeed, this should apply to all classes, with policies that guarantee work security, health insurance, and severance pay for those who encounter job instability. This would ensure that all workers are able to overcome this crisis in a stable way.

In closing, we acknowledge the need for the Korean government and specific companies to develop disaster management schemes and customize them so as to ensure that the working environments of each class are protected during future national disasters.

Although we present relevant contributions to the literature, we must still identify the limitations of our study and concomitantly provide suggestions for subsequent studies on the topic. First, this study was conducted at a time in which the COVID-19 crisis had entered a stable phase; thus, in the future, studies should examine our interest variables by these new classes of occupation at a point at which a virus is rapidly spreading. Second, the Korean government imposed few restrictions on leisure activities at a national level owing to the Korean population’s voluntary adherence to social distancing [26]; moreover, to the best of our knowledge, there are no investigations on which activities within the workplace are vulnerable to the infection. This is important information, as participation in leisure activities is growing in the country as of right now [58]. Thus, future studies should provide such data, as it will allow for the examination of more types of workplaces, thereby generating more meaningful and holistic scientific findings.

## Figures and Tables

**Table 1:** Differences between the new classification of the occupations owing to the COVID-19 pandemic (as proposed by Reich [4], based on the United States of America context) and the adapted classifications proposed for the Korean setting.

New Classes of Occupation Owing to the COVID-19 Pandemic Based on the USA Setting		New Classes of Occupation Owing to the COVID-19 Pandemic Adapted to the Korean Setting	
The Remotes	Professional, senior, and technical workers who work in their own space without changes during the pandemic	The Remotes	Workers who can work from home without wage changes (at least 2 days per week) during the pandemic
The Essentials	Nurses, pharmacy staff, police officers, firefighters, and general office workers who work only in their own space and are essential in dealing with the pandemic	The Essentials-1	Workers who work as before without wage changes during the pandemic
		The Essentials-2	Workers who work shorter hours or every other day without wage changes during the pandemic
The Unpaid	Workers who go to work same as before, but receive reduced wages or face unpaid leave; whose workplaces have temporarily closed; who are placed on standby; or who do not work during the pandemic	The Unpaid-1	Workers who go to work the same as before, but receive reduced wages during the pandemic
		The Unpaid-2	Workers who work shorter hours or work every other day, but receive reduced wages during the pandemic
The Forgotten	People for whom social distancing during the pandemic is practically impossible, such as prison inmates, people in immigration detention centers, or migrant farm worker camps, Native American sanctuaries, and homeless people facilities	The Forgotten	Workers on unpaid leave; whose workplaces have temporarily closed; who have been placed on standby; or who do not work during the pandemic

**Table 2:** Participants’ occupational calling, psychological health, and quality of life according to their sociodemographic characteristics (*N* = 1029).

Variable	*n* (%)	Occupational Calling	F/t	Post Hoc Test	Psychological Health	F/t	Post Hoc Test	QoL	F/t	Post Hoc Test
		M ± SD			M ± SD			M ± SD		
**Gender**										
Male	546 (53.1)	31.20 ± 4.97			22.13 ± 7.04			31.50 ± 8.59		
Female	483 (46.9)	29.92 ± 5.12	16.44 ***		23.42 ± 6.94	8.73 ***		29.02 ± 8.51	21.50 ***	
Total	1029	30.60 ± 5.08			22.74 ± 7.02			30.34 ± 8.64		
**Age**										
20–29 years ^a^	68 (6.6)	30.37 ± 5.45			22.74 ± 8.27			30.43 ± 10.38		
30–39 years ^b^	559 (54.3)	29.96 ± 5.24			22.86 ± 6.89			30.64 ± 8.40		
40–49 years ^c^	289 (28.1)	31.34 ± 4.69	6.06 ***	b > c, d	22.70 ± 6.82			29.73 ± 8.75		
50–59 years ^d^	108 (10.5)	32.02 ± 4.37			22.24 ± 7.36			30.19 ± 8.48		
60 years or higher ^e^	5 (.5)	31.80 ± 6.61			22.00 ± 9.41			33.40 ± 5.77		
Total	1029	30.60 ± 5.08			22.74 ± 7.02			30.34 ± 8.64		
**Marital Status**										
Single ^1^	468 (45.5)	29.20 ± 5.14			23.63 ± 7.26			29.11 ± 9.11		
Married ^2^	543 (52.8)	31.56 ± 4.83	21.46 ***	1 > 2	21.96 ± 6.68	7.24 **	1 > 2	31.40 ± 8.12	9.01 ***	1 > 2
Widowed ^3^	18 (1.7)	30.28 ± 5.15			22.94 ± 8.21			30.22 ± 7.67		
Total	1029	30.60 ± 5.08			22.74 ± 7.02			30.34 ± 8.64		
**Monthly Income**										
<2 million KRW ^α^	160 (15.5)	28.96 ± 4.94			24.85 ± 7.76			27.13 ± 9.80		
2–3.99 million KRW ^β^	554 (53.8)	30.10 ± 5.03			22.85 ± 6.71			30.03 ± 8.34		
4–5.99 million KRW ^γ^	202 (19.6)	31.99 ± 4.66			21.81 ± 6.64			32.01 ± 7.84		
6–7.99 million KRW ^δ^	62 (6.0)	32.47 ± 4.65	13.35 ***	α > β, γ, δ, ε, ζ	20.98 ± 7.73	5.55 ***	α > γ, δ >	32.61 ± 7.18	9.66 ***	α > β, γ, δ, ε, ζ
8–9.99 million KRW ^ε^	33 (3.2)	33.03 ± 5.36			21.48 ± 6.15			33.85 ± 8.54		
≥10 million KRW ^ζ^	18 (1.7)	34.33 ± 4.79			19.39 ± 8.22			35.39 ± 9.92		
Total	1029	30.60 ± 5.08			22.74 ± 7.02			30.34 ± 8.64		
**Type of Leisure**										
Watching cultural and art activities	562 (54.6)	31.10 ± 5.23			22.44 ± 6.97			30.51 ± 8.91		
Participating in cultural and art activities	142 (13.8)	32.01 ± 5.08			22.86 ± 6.32			32.46 ± 8.44		
Watching sports	421 (40.9)	31.00 ± 5.16			22.45 ± 6.94			30.79 ± 8.61		
Participating in sports	225 (21.9)	30.94 ± 5.25			22.88 ± 6.87			31.20 ± 8.62		
Going on tourism activities	514 (50.0)	31.19 ± 5.15			22.39 ± 7.08			30.85 ± 8.73		
Enjoying hobbies and entertainment	601 (58.4)	30.83 ± 5.37			22.45 ± 7.41			30.46 ± 9.24		
Resting (e.g., sleeping, listening to music, taking a siesta, drinking tea)	891 (86.6)	30.51 ± 5.06			22.71 ± 7.06			30.14 ± 8.60		
Participating in social and other activities	324 (31.5)	31.10 ± 5.22			22.60 ± 7.00			29.91 ± 8.38		

KRW: Korean Won; QoL: Quality of life. M: Mean; SD: Standard Deviation; F: An F statistic is a value you get when you run an ANOVA test or a regression analysis to find out if the means between two populations are significantly different. t: In T-test, we measure how far is “the difference between two means” from “the null value”. While in ANOVA, we measure the difference (variability) between the groups; a: 20–29 years; b: 30–39 years; c: 40–49 years; d: 50–59 years; e: 60 years or higher; 1: Single; 2: Married; 3: Widowed; α: <2 million KRW; β: 2–3.99 million KRW; γ: 4–5.99 million KRW; δ: 6–7.99 million KRW; ε: 8–9.99 million KRW; ζ: ≥10 million KRW; *** *p* < 0.001, ** *p* < 0.01.

**Table 3:** Correlations between COVID-19 new occupational class, calling, psychological health, and QoL (*N* = 1029).

	1	2	3	4
the new classes of occupation owing to the COVID-19 pandemic	1		1	
Psychological health	0.191 **	1		
QoL	−0.175 **	−0.703 **	1	
Occupational Calling	0.554	−0.370 **	0.505 **	1

QoL: Quality of life; ** *p* < 0.01.

**Table 4:** Participants’ calling, psychological health, and QoL according to our Korean-adapted version of the new classes of occupation owing to the COVID-19 pandemic (*N* = 1029).

Variable	*n* (%)	Occupational Calling	F/t	Post Hoc Test	Psychological Health	F/t	Post Hoc Test	QoL	F/t	Post Hoc Test
		M ± SD			M ± SD			M ± SD		
The Remotes ^ⅰ^	68 (6.6)	32.37 ± 5.40			20.91 ± 7.16			32.71 ± 9.02		
The Essentials-1 ^ⅱ^	657 (63.8)	30.32 ± 5.05			22.11 ± 6.74			30.97 ± 8.15		^ⅰ^^>^^ⅴ^^,^^ⅵ^
The Essentials-2 ^ⅲ^	53 (5.2)	31.19 ± 5.12			22.87 ± 5.44		^ⅰ^^>^^ⅴ^^,^^ⅵ^	32.13 ± 8.68		^ⅱ^^>^^ⅴ^^,^^ⅵ^
The Unpaid-1 ^ⅳ^	108 (10.5)	31.62 ± 4.34	3.53 **		23.61 ± 7.15	7.98 ***	^ⅱ^^>^^ⅴ^^,^^ⅵ^	28.42 ± 8.42	7.54 ***	^ⅲ^^>^^ⅴ^
The Unpaid-2 ^ⅴ^	68 (6.6)	29.75 ± 5.79			25.68 ± 7.70			26.90 ± 9.60		
The Forgotten ^ⅵ^	75 (7.3)	30.31 ± 4.90			25.89 ± 7.80			27.27 ± 9.89		
Total	1029	30.60 ± 5.08			22.74 ± 7.02			30.34 ± 8.64		

QoL: Quality of life; M: Mean; SD: Standard Deviation; F: An F statistic is a value you get when you run an ANOVA test or a regression analysis to find out if the means between two populations are significantly different. t: In T-test, we measure how far is “the difference between two means” from “the null value”. While in ANOVA, we measure the difference (variability) between the groups; *** *p* <0.001, ** *p* <0.01.

## References

[1] Zhang S.X., Wang Y., Rauch A., Wei F. (2020). Unprecedented disruption of lives and work: Health, distress and life satisfaction of working adults in China one month into the COVID-19 outbreak. Psychiatry Res..

[2] Montenovo L., Jiang X., Rojas F.L., Schmutte I., Simon K., Weinberg B., Wing C. (2020). Determinants of Disparities in Covid-19 Job Losses. National Bureau of Economic Research.

[3] Germani A., Buratta L., Delvecchio E., Mazzeschi C.P. (2020). Emerging Adults and COVID-19: The Role of Individualism-Collectivism on Perceived Risks and Psychological Maladjustment. Int. J. Environ. Res. Public Health.

[4] Reich R. Coronavirus Outbreak Covid-19 Pandemic Shines a Light on a New Kind of Class Divide and its Inequalities. The Guardian. https://www.theguardian.com/commentisfree/2020/apr/25/covid-19-pandemic-shines-a-light-on-a-new-kind-of-class-divide-and-its-inequalities.

[5] Choi B.J. The Future of E-Government after Corona 19 Electronic Newspaper. https://www.etnews.com/20200421000297.

[6] Guerrero L.R., Avgar A.C., Phillips E., Sterling M.R. (2020). They are Essential Workers Now, and Should Continue to Be: Social Workers and Home Health Care Workers during COVID-19 and Beyond. J. Gerontol. Soc. Work.

[7] Choi Y.J. Telecommuting from Home, Commuting to Each Other… The Form of Work Changed to Corona 19. Yunhap News. https://www.yna.co.kr/view/AKR20200227107800003?input=1195m.

[8] Kim S.Y. “If you Hate Unpaid Leave, Quit Now”… We Cry Twice in Contracts and Special Employment Jobs, ‘Corona Gap’. World Daily. http://www.segye.com/newsView/20200430514092?OutUrl=naver.

[9] Albright V., Borman W.C., Hedge J.W. (2012). Workforce Demographics in the United States. The Oxford Handbook of Work and Aging.

[10] Hunter I., Dik B.J., Banning J.H. (2010). College students’ perceptions of calling in work and life: A qualitative analysis. J. Vocat. Behav..

[11] Um D.H., Kim J.S., Lee H.W., Lee S.H. (2017). Psychological effects on medical doctors from the middle east respiratory syndrome (MERS) outbreak: A comparison of whether they worked at the MERS occurred hospital or not, and whether they participated in MERS diagnosis and treatment. J. Korean Neuropsychiatr. Assoc..

[12] Lee S.Y. (2015). Counterattack of MERS, unexpected confusion. Doctorsnews.

[13] Collin S.-O. Duty Governing Human Actions: To Act Because it is Righteous. https://papers.ssrn.com/sol3/papers.cfm?abstract_id=3560203.

[14] Cho H.S. 480,000’ Employees’ in April… the Worst’ Employment Tragedy’ after a Foreign Exchange Crisis. Korea Economics tv. http://www.wowtv.co.kr/NewsCenter/News/Read?articleId=A202005130069&t=NNv.

[15] Byeon B.S. Korea-Hong Kong Workers Announce Joint Declaration of KCTU-HKCTU (HKCTU) as an International Solidarity Key to Respond to Pandemic Corona19. Labor and the World. http://worknworld.kctu.org/news/articleView.html?idxno=251473.

[16] Oh J., Lee J.-K., Schwarz D., Ratcliffe H.L., Markuns J.F., Hirschhorn L.R. (2020). National response to COVID-19 in the Republic of Korea and lessons learned for other Countries. Health Syst. Reform.

[17] Rajkumar R.P. (2020). COVID-19 and mental health: A review of the existing literature. Asian J. Psychiatry.

[18] Parnell D., Widdop P., Bond A., Wilson R. COVID-19, Networks and Sport. Manag. Sport Leis..

[19] Lesser I., Nienhuis C.P. (2020). The Impact of COVID-19 on Physical Activity Behavior and Well-Being of Canadians. Int. J. Environ. Res. Public Health.

[20] Wilson C.A., Britt T.W. Living to Work: The Role of Occupational Calling in Response to Challenge and Hindrance Stressors. Work Stress.

[21] Keller A.C., Spurk D., Baumeler F., Hirschi A. (2016). Competitive climate and workaholism: Negative sides of future orientation and calling. Pers. Individ. Differ..

[22] Bunderson J.S., Thompson J.A. (2009). The call of the wild: Zookeepers, callings, and the double-edged sword of deeply meaningful work. Adm. Sci. Q..

[23] Alon T., Doepke M., Olmstead-Rumsey J., Tertilt M. (2020). The Impact of COVID-19 on Gender Equality. National Bureau of Economic Research 2020.

[24] Sion G. (2020). COVID-19 and Labour Law: Republic of Korea. Ital. Labour Law e-J..

[25] Kim C.S. 26.3% of Non-Regular Workers Experience ‘Corona 19 Unemployment’… More than Six Times the Regular Job. Yunhap News. https://www.yna.co.kr/view/AKR20200622059700004?input=1195m.

[26] Aum S., Lee S.Y., Shin Y. (2020). COVID-19 Doesn’t Need Lockdowns to Destroy Jobs: The Effect of Local Outbreaks in Korea. National Bureau of Economic Research.

[27] Ha Y.J., Choi Y.U., Eun H.Y., Sohn Y.W. (2014). Validation of the Korean version of Multidimensional Calling Measure(MCM-K). Korean J. Ind. Organ. Psychol..

[28] Hagmaier T., Abele A.E. (2012). The multidimensionality of calling: Conceptualization, measurement and a bicultural perspective. J. Vocat. Behav..

[29] Goldberg D. (1978). Manual of the General Health Questionnaire, NFER.

[30] Chang S.J. (2000). Standardization of collection and measurement of health statistics data. The Korean Society for Preventive Medicine.

[31] Higgs P., Hyde M., Wiggins R., Blane D. (2003). Researching Quality of Life in Early Old Age: The Importance of the Sociological Dimension. Soc. Policy Adm..

[32] Allison J.A., Wrightsman L.S. (1993). Rape: The Misunderstood Crime.

[33] Soh C.-H.S. (1993). Sexual equality, male superiority, and Korean women in politics: Changing gender relations in a patriarchal democracy. Sex Roles.

[34] Baryła-Matejczuk M., Skvarciany V., Cwynar A., Poleszak W., Cwynar W. (2020). Link between Financial Management Behaviours and Quality of Relationship and Overall Life Satisfaction among Married and Cohabiting Couples: Insights from Application of Artificial Neural Networks. Int. J. Environ. Res. Public Health.

[35] Kim H.-S., Kim S.-S. (2017). A converged study about influences of job stress, job security, depression, family bond, subjective health status, social support on quality of life in married middle-aged male. J. Korea Converg. Soc..

[36] Lee G.A. Pohang Medical Center Nurse Corona19 You Were Afraid and Quit? Don’t Ignore the Mission of Your Colleagues. The Seoul Newspaper, the Latest in Society. https://www.seoul.co.kr/news/newsView.php?id=20200303500155&wlog_tag3=naver.

[37] Delgado D., Quintana F.W., Perez G., Liprandi A.S., Ponte-Negretti C., Mendoza I., Baranchuk A. (2020). Personal Safety during the COVID-19 Pandemic: Realities and Perspectives of Healthcare Workers in Latin America. Int. J. Environ. Res. Public Health.

[38] Borst R.T., Lako C.J. (2017). Proud to be a public servant? An analysis of the work-related determinants of professional pride among dutch public servants. Int. J. Public Adm..

[39] Kim H.G., Kim C.S. (2017). An Empirical Measurement and Analysis of the Influence of Public Officials’ Personality on Public Service: With as Central Professional Soldiers. J. Korean Policy Stud..

[40] Carmassi C., Gesi C., Simoncini M., Favilla L., Massimetti G., Olivieri M.C., Conversano C., Santini M., Dell’Osso L. (2016). DSM-5 PTSD and posttraumatic stress spectrum in Italian emergency personnel: Correlations with work and social adjustment. Neuropsychiatr. Dis. Treat..

[41] Carmassi C., Gesi C., Corsi M., Cremone I.M., Bertelloni C.A., Massimetti E., Olivieri M.C., Conversano C., Santini M., Dell’Osso L. (2018). Exploring PTSD in emergency operators of a major University Hospital in Italy: A preliminary report on the role of gender, age, and education. Ann. Gen. Psychiatry.

[42] Jang S.G. The Catastrophe of COVID-19 INFECTION and the Role of Experts. Council Newspaper. http://www.doctorsnews.co.kr/news/articleView.html?idxno=133636.

[43] Park E.H. (2020). The Effects of professional Consciousness on Calling, Communication Competence, Job Satisfaction of Caregivers. J. Korea Converg. Soc..

[44] Cho S.H. 4th Week without Hell and Dinner. Can I Keep Working from Home? Money Today. https://news.mt.co.kr/mtview.php?no=2020030916054046225.

[45] Lee D., Kim J.-Y., Kang H.-S. (2016). The Emotional Distress and Fear of Contagion Related to Middle East Respiratory Syndrome(MERS) on General Public in Korea. Korean J. Psychol. Gen..

[46] Chitra T., Karunanidhi S. The Impact of Resilience Training on Occupational Stress, Resilience, Job Satisfaction, and Psychological Well-Being of Female Police Officers. J. Police Crim. Psychol..

[47] Stefano T., Balducci C. (2018). Stress-preventive management competencies, psychosocial work environments, and affective well-being: A multilevel, multisource investigation. Int. J. Environ. Res. Public Health.

[48] June K.J., Choi E.S., Park M.-J. (2013). Effect of Psychosocial Work Environment and Self-efficacy on Mental Health of Office Workers. Korean J. Occup Health Nurs..

[49] Shacham M., Hamama-Raz Y., Kolerman R., Mijiritsky O., Ben-Ezra M., Mijiritsky E. (2020). COVID-19 Factors and Psychological Factors Associated with Elevated Psychological Distress among Dentists and Dental Hygienists in Israel. Int. J. Environ. Res. Public Health.

[50] Song D.S., Song J.H. “I’m Taking a Break from This Month…” Corona Restructuring Wind. No Cut News-No Cut Economy. https://www.nocutnews.co.kr/news/5339114.

[51] Shockey T., Zack M., Sussell A. (2017). Health-related quality of life among US workers: Variability across occupation groups. Am. J. Public Health.

[52] Han X., Juanle W., Zhang M., Wang X. (2020). Using Social Media to Mine and Analyze Public Opinion Related to COVID-19 in China. Int. J. Environ. Res. Public Health.

[53] Ministry of Trade, Industry and Energy A Public-Private Joint Support System is in Operation to Respond to Corona 19 Related Corporate Difficulties. Policy Briefing. http://www.korea.kr/news/pressReleaseView.do?newsId=156376912.

[54] Jeon J. [I like Korea] From Masks to Rice Noodles and Bread… Food Industry Connecting Donation Relays. Daily Report. http://www.m-i.kr/news/articleView.html?idxno=687697.

[55] Oh S.I., Cho J.H. (2016). Sociological Reflection on the Decrease of the Quality of Life in Korea: Focusing on the Uncertainty of Neoliberal Labor Markets.

[56] Kalyaev V., Salimon A.I., Korsunsky A.M., Denisov A.A. (2020). Fast mass-production of medical safety shields under COVID-19 quarantine: Optimizing the use of University fabrication facilities and volunteer labor. Int. J. Environ. Res. Public Health.

[57] Tran B.X., Dang A.K., Thai P.K., Le H.T., Le X.T.T., Do T.T.T., Nguyen T.H., Pham H.Q., Phan H.T., Vu G.T. (2020). Coverage of Health Information by Different Sources in Communities: Implication for COVID-19 Epidemic Response. Int. J. Environ. Res. Public Health.

[58] Kim Y.-J., Cho J.-H. (2020). Correlation between Preventive Health Behaviors and Psycho-Social Health Based on the Leisure Activities of South Koreans in the COVID-19 Crisis. Int. J. Environ. Res. Public Health.

